# Some Causes of Febrile Disturbance in Women during the Lying-In Period

**Published:** 1894-12-01

**Authors:** George Drummond Robinson

**Affiliations:** Obstetric Physician to St. George's and St. James's Dispensary


### Medical Progress and Hospital Clinics.

[*The Editor will be glad to receive offers of co-operation and contributions from members of the profession. All letters should be addressed to* The Editor, The Lodge, Porchester Square, London, W.]

### "SOME CAUSES OF FEBRILE DISTURBANCE, IN WOMEN DURING THE LYING-IN PERIOD."

By George Drummond Robinson, M.D. and B.S. (London), M.R.C.P., Obstetric Physician to St. George's and St. James's Dispensary.

It has long been known that, during the lying-in period, the whole organism is in such a condition that a cause, trivial enough as regards its ultimate results, and perhaps hardly noticeable under other conditions, may produce a marked rise of temperature, accompanied, perhaps, with other alarming symptoms.

Apart, then, from the more serious causes of fever during the puerperal period, directly connected with the pelvic organs, such as septic infection (septicæmia, pyæmia), septic intoxication (sapræmia), and localised pelvic inflammations (perimetritis and parametritis), there are others. Some of these depend only indirectly, others not at all, on the processes of pregnancy and parturition, but all are capable of causing considerable anxiety to the practitioner.

**Loaded Intestines.**—That the accumulation of fæcal material in the intestine may occasionally lead to profound constitutional disturbance, even in previously healthy subjects, is undoubted. Thus a young married woman was suddenly seized with abdominal pain and profound collapse whilst engaged in her household duties. A tumour was discovered in the right iliac fossa, and a ruptured extra-uterine pregnancy was suspected. Some hours later a very copious action of the bowels took place, and the patient rapidly recovered from her condition of shock. The abdominal tumour (fæcal) had disappeared. More often, during the puerperal period, fæcal retention gives rise to febrile symptoms.

Even a free action of the bowels does not exclude this cause, for it must be remembered that most, if not all, women suffer from fæcal retention during pregnancy, and it may need repeated actions of the bowels, after delivery, to remove the accumulation completely. It is sometimes surprising, under these circumstances, to find how copious the motions are, even after several evacuations. The administration of a purge regularly for some days after delivery is a useful routine practice, and this simple proceeding will prevent, or cure, not a few cases of fever during the puerperal state.

**Injudicious Diet** is another important and well-known cause of fever in the lying-in woman, of which it is only necessary to make passing mention.

**Lung Disease,** most frequently phthisis, is, in my experience, a not very infrequent cause of puerperal temperature. Pregnancy and parturition usually incite tubercular processes, and especially phthisis, to increased activity. It is well, therefore, carefully to examine the lungs of any patient who, during the puerperal period, is found to be suffering from a febrile temperature, for which there is no very obvious cause.

**The Specific Fevers** should always be thought of as among the less common causes of pyrexia occurring after labour, and we must all have seen instances of one or other of these diseases developing during the first few days after confinement.

Amongst the most common to occur, and for which we must be on the look-out, are enteric and scarlet fevers; the latter, be it remembered, presenting the ordinary characters of scarlet fever, and not, as was formerly taught, manifesting itself as puerperal septicæmia. More rarely rheumatic fever, frequently of a severe type, attacks the lying-in woman, sometimes also pneumonia, small-pox, and others of the specific fevers.

**Tonsillitis** is another not uncommon cause of fever following labour. As the temperature is often high and the constitutional disturbance severe, these cases may be very puzzling if the patients make no complaint of throat symptoms, and this is frequently the case even when they are closely questioned on the point.

Having once been much perplexed over such a case, I always now make a routine practice of examining the tonsils whenever I meet with a rise of temperature of doubtful cause during the puerperal period, and I have been surprised to find how frequently tonsillitis occurs in lying-in women of the hospital class.

**Defective Sanitary Arrangements,** permitting the air to become contaminated with sewer gas, &c., are no doubt responsible for many of these cases of tonsillitis.

Even more severe forms of pyrexial disturbance in childbed have been attributed to this cause, and some maintain that many of those fatal cases of "puerperal fever," in which no evidence of direct infection can be obtained, can be traced to this source. Such cases, for instance, as those in which no vaginal examination has been made either before or after labour.

**The Breasts** are frequent and important causes of puerperal temperature.

The mammary abscess, with its marked symptoms, both local and general, is not easily overlooked.

There is, however, one condition of the breasts that leads to constitutional disturbance (including pyrexia), as marked as that of mammary abscess that might on casual examination be undetected on account of the slightness of the local symptoms it frequently presents.

The common cracked nipple may be so exquisitely tender that sometimes the mother refrains from putting the child to the diseased nipple, and the breast consequently becomes engorged with milk. This may give rise to striking phenomena, which a single example will illustrate. A patient, a week after labour, was seized with headache, pains in the limbs, and profound general malaise. The temperature was 105·8 degrees. She looked very ill, pulse and respiration were rapid, the skin hot, the tongue foul. There was no obvious cause for the condition. Questions regarding the state of the breasts gave no assistance. The mammæ were, however, examined, and one was found tense and somewhat tender, and the patient then confessed that the child had been denied the breast on account of a sore nipple. Removal of the superfluous milk by means of a breast pump immediately lowered the temperature, and the other distressing symptoms promptly disappeared. Milk fever or ephemeral fever is now known not to depend on the breasts, but probably to some slight absorption of chemical poison from the wounded genital canal.

**Eclampsia** is another, fortunately rare, cause of puerperal temperature.

In diagnosing the cause of fever in a woman recently delivered, besides considering the question of sepsis in its various degrees, it is well therefore to adopt a routine plan of investigation. The condition of the alimentary tract, including the tonsils, should be carefully gone into, also that of the respiratory organs and the breasts. Defects in the sanitary arrangements of the lying-in chamber should be sought for, and a watch kept for evidence of the presence of the specific fevers.

